# Growing Season Temperatures in Europe and Climate Forcings Over the Past 1400 Years

**DOI:** 10.1371/journal.pone.0009972

**Published:** 2010-04-01

**Authors:** Joel Guiot, Christophe Corona, ESCARSEL members

**Affiliations:** 1 CEREGE (European Centre of Research and Teaching in Geosciences of Environment), UMR 6635 Aix-Marseille University/CNRS, Aix-en-Provence, France; 2 ECCOREV (Continental Ecosystems and Risk related to Environment), FR 3098 Aix-Marseille University/CNRS, Aix-en-Provence, France; Centre National de la Recherche Scientifique, France

## Abstract

**Background:**

The lack of instrumental data before the mid-19th-century limits our understanding of present warming trends. In the absence of direct measurements, we used proxies that are natural or historical archives recording past climatic changes. A gridded reconstruction of spring-summer temperature was produced for Europe based on tree-rings, documentaries, pollen assemblages and ice cores. The majority of proxy series have an annual resolution. For a better inference of long-term climate variation, they were completed by low-resolution data (decadal or more), mostly on pollen and ice-core data.

**Methodology/Principal Findings:**

An original spectral analog method was devised to deal with this heterogeneous dataset, and to preserve long-term variations and the variability of temperature series. So we can replace the recent climate changes in a broader context of the past 1400 years. This preservation is possible because the method is not based on a calibration (regression) but on similarities between assemblages of proxies. The reconstruction of the April-September temperatures was validated with a Jack-knife technique. It was also compared to other spatially gridded temperature reconstructions, literature data, and glacier advance and retreat curves. We also attempted to relate the spatial distribution of European temperature anomalies to known solar and volcanic forcings.

**Conclusions:**

We found that our results were accurate back to 750. Cold periods prior to the 20^th^ century can be explained partly by low solar activity and/or high volcanic activity. The Medieval Warm Period (MWP) could be correlated to higher solar activity. During the 20^th^ century, however only anthropogenic forcing can explain the exceptionally high temperature rise. Warm periods of the Middle Age were spatially more heterogeneous than last decades, and then locally it could have been warmer. However, at the continental scale, the last decades were clearly warmer than any period of the last 1400 years. The heterogeneity of MWP versus the homogeneity of the last decades is likely an argument that different forcings could have operated. These results support the fact that we are living a climate change in Europe never seen in the past 1400 years.

## Introduction

The lack of instrumental data before the mid 19th century limits our ability to interpret the present warming trend. The second half of the 20^th^ century was very likely (a probability >90% according to the Intergovernmental Panel on Climate Change (IPCC) definition) to be caused by man in the comparison with previous natural changes over the past 500 years [1]. The IPCC concluded also that the second half of the 20^th^ century had a probability >66% to be warmer than any period in the past 1300 years. These conclusions were based on parameters measured on natural archives recording past climatic changes–also called proxies–such as tree-rings, corals, ice cores, pollen series and historical documentary data [2], [3], [4]. These reconstructions demonstrate that the recent warming trend is exceptional. They also support a scenario where the Medieval Warm Period (MWP) was followed by a long cooler period starting after 1400 and ending at the beginning of the 20^th^ century. This cooler period is generally called the Little Ice Age (LIA) but there is some vagueness in its extent (for example [4] show a clear cooling after A.D 1400 and Esper et al (2002) at about 1550). Although most of the 20^th^ century could be comparable to the MWP, temperatures over the past decade clearly exceeded this period. Reconstruction amplitudes also varied widely with little agreement across this time period. These problems have been extensively examined, and may be related to the methodology and quality of datasets used [5], [6], [7], [8], [9], [10], [11]. According to modeling results, these long-term changes have been related to volcanic, solar and anthropogenic forcings [12].

The purpose of this work was to place recent warming trend, demonstrated with instrumental series, in the larger context of millennial changes. To this end, it was necessary to ensure correct representation of different regions examined; for example, inter-tropical regions are always under-represented in global analyses [2]. This paper focuses exclusively on Europe because we think an adequate balance is reached only by working continent-to-continent. It is also essential to deal carefully with low frequency variations. [5] showed that the low frequency behavior of tree-ring series can cause an underestimation of MWP levels in temperate regions. This, in turn, would lead to an over-emphasis on recent warming. To address this challenge, [5] proposed to standardize the tree-ring series in a way that preserves climatic low frequencies. An alternative solution [4] uses tree-ring series to estimate high frequency climate variations, and applies ice cores and lake/oceanic sediment proxies to reconstruct low frequency variations. The cut-off period was 80 years in this case. However, [4] did not solve the proxy calibration problem because proxies were just rescaled in temperature units and assumed to record the same climatic signal. This latter assumption is problematic: according to [13], methodological issues have not been fully resolved regarding the potential underestimation of long-term variability in methods such as climate-field reconstruction or proxy scaling. In this paper, we propose an alternative method adapted from [14] that is based on a decomposition of spectral characteristics of various proxies into three bands. The reconstruction must preserve the observed temperature variance so that comparisons of the reconstructed to the observed climate variability is meaningful. Our aim is to show that this alternative method can reduce amplitude losses significantly. Finally, we compared recent warming trends to MWP temperature and study the main climate variations under the light of solar, volcanic and/or anthropogenic forcings.

## Results and Discussion

**Figure 1:**
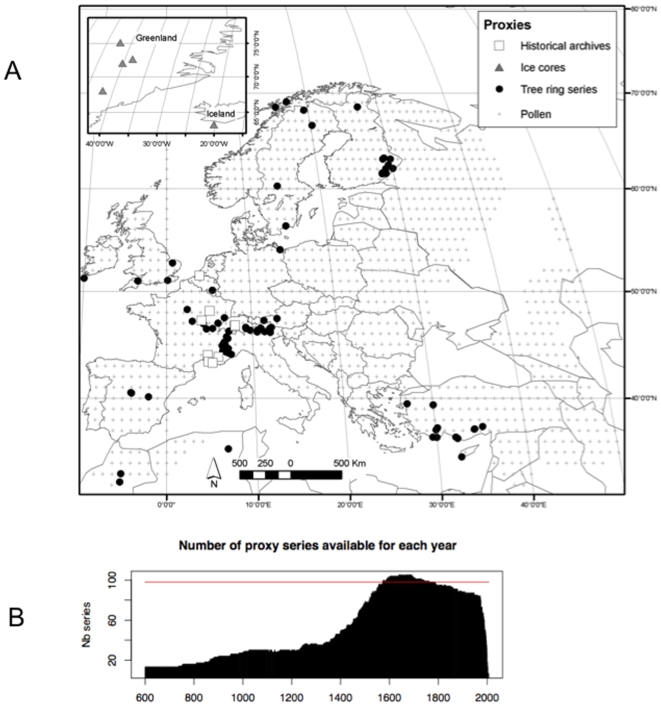
Characteristics of the proxies. A) Map of the proxies used. B) Number of proxy series available as a function of the year, from 600 to 2007.

**Figure 2:**
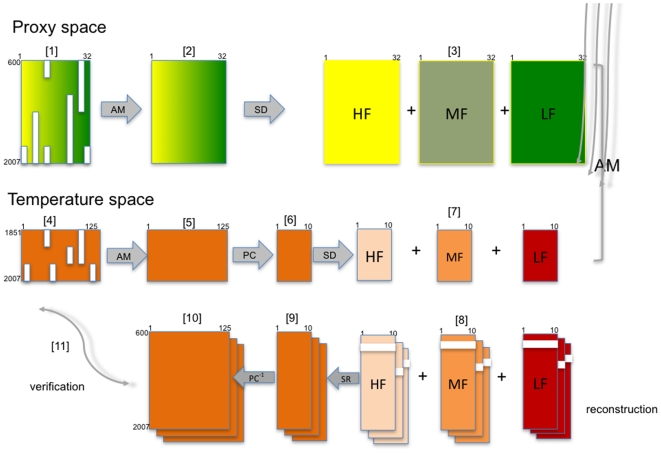
Schema of the reconstruction method (see text for more details). Abbreviations: AM, analog method, SD, spectral decomposition, PC, transformation into principal components, SR, spectral recomposition, PC^−1^, back-tranformation of the principal components into original variables, HF, high frequency, MF, medium frequency, LF, low frequency.

First, the data are presented: proxy data (Fig. 1) used to reconstruct the growing season temperature, and data used for validation. Then the results are analyzed and compared to previous reconstructions and forcing data (solar and volcanism activities). The method is described in a simplified way in Fig. 2 and in an exhaustive way in the appendix.

### 1. Proxy data

To achieve these results, a large proxy series dataset was compiled from public databases or collaborations. These data were mainly tree-ring width series, indexed climate series based on historical written documents, ice-core isotopic series and pollen-based series. Table S1 and Fig. 1A present the nature and geographical position of these series. The 95 tree-ring series extended through various portions of the 600–2000 period, with maximum density noted after 1200. These data have been indexed using the adaptative regional growth curve method [15] (see a few examples in Fig. S1). They were complemented with 16 historical series of indices calculated by various authors, for Southern France, Switzerland and the European Low Countries. Isotopic series measured in ice cores of Greenland have lower resolution, of at most 5–10 years. Still lower the resolution of annual temperatures reconstructed from pollen data [16] is with a 100 years time-step. The spatial resolution of pollen data is 1° longitude and latitude. All low resolution series were interpolated at the annual time-step, using a two- dimension spline function (function splint of R-package “fields” version 6.0.1, http://www.image.ucar.edu/Software/Fields). Fig. 1B displays proxy number available for each year. A decline in proxy numbers was observed before 1400.

### 2. Climatic data

The monthly temperature series used for calibration have been downloaded from the Hadley Center - Climatic Research Unit dataset HADCRUT3 (http://www.cru.uea.ac.uk/cru/data/temperature/, [17]). They cover the period of 1850–2007 at a resolution of 5° longitude and latitude. As most proxies are sensitive to the growing season temperature, we decided to take the averaged monthly temperature from April to September. They are expressed as anomalies relative to the 1961–1990 average. These data have a large number of gaps. The choice of climatic variable may be discussed. We know that some tree species, especially those in southern Europe, are more sensitive to precipitation [18], but summer can also stress tree-growth. Therefore, gridded approaches, as applied here, make it necessary to compromise and choose the most representative climatic factors.

### 3. Validation and forcing data

To validate the temperature reconstruction, we used the gridded monthly temperatures from 1766 to 2000 reconstructed by [19] and based on long instrumental series of Europe (http://www.ncdc.noaa.gov/paleo/pubs/casty2007/casty2007.html). The spatial resolution is 0.5° in latitude and longitude. We also used reconstructed gridded temperatures based on various proxies (e.g. documentary sources, tree-rings…) that extended back to 1659 [20].

Although they are not only caused by summer temperature, but also by also winter precipitation and solar irradiation [21], and although uncertainties remain about the time lag in response, however, glacier advances and retreats correlate generally well with long-term temperature variations. They are the only proxies of summer temperatures that cover the last two millennia and are independent of proxies included in our reconstructions. The Great Aletsch glacier is the largest and most documented glacier in the Alps. A long history of glacier advances and retreats is available from [22] and is supported by radiocarbon and tree-ring dating, moraine investigations and annual measurements since 1892. This curve was compared with our smoothed reconstruction of summer temperature from the closest grid-point. Another long series, with a sufficient resolution is available for Southern Scandinavia [23] and was compiled from various papers [24], [25], [26], [27].

Volcanic eruptions are responsible for cooling of the earth, mainly in the years immediately following their occurrence [28], [29]. We studied the effects of volcanic forcing on our reconstruction by selecting the coldest of three years starting from the peak in the weighted dust veil index series (http://www.ncdc.noaa.gov/paleo/ei/ei_data/volcanic.dat) [30] and volcanic radiative forcing curves [29]. To study the effects of solar activity on temperatures, we used solar activity data reconstructed from residual radiocarbon and beryllium-10 isotopes [31].

### 4. Comparison with spatial reconstructions

The CASTY dataset [19] is totally independent of our reconstruction, because these authors did not use any proxies, but includes about 150 climate stations monitored during the 20^th^ century. The number declines exponentially through the 19^th^ century, and reaches less than 30 in the 18^th^ century. As such the CASTY dataset is of a poorer quality before 1800. Because of this limitation in spatial coverage back in time, particularly at higher elevations, the weight of single stations is increased [32].

**Figure 3:**
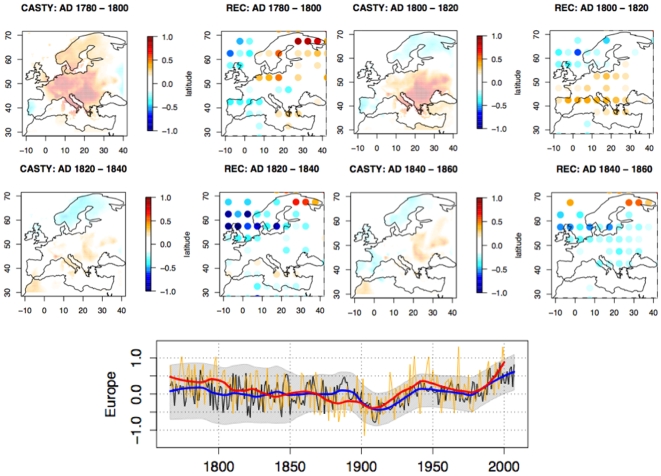
Comparison of the reconstruction of the April to September temperature anomalies (this paper) with the one produced by [19]. The above panel presents maps for several time periods. The CASTY maps have a 0.5° resolution while the reconstruction in this paper has a resolution of 5°. The lower panel show the mean temperature for Europe: in orange, the CASTY series (trend in red) and in black, this paper's reconstruction (trend in blue) with (in grey) the 90% confidence interval.

Maps of our reconstructions were averaged using 20-year windows and compared to equivalent CASTY maps adapted to the resolution of our reconstructed maps (Fig. 3). These comparisons were quantified by the square-root of the mean squared discrepancies (RMSD) between the maps on the common grid-points (Table 1). For the period of 1780–1800, the CASTY data indicate elevated temperatures everywhere except on Iberian peninsula and Eastern Europe. Our reconstruction appears too cold in the United Kingdom and, for some grid-points in Central Europe. For the 1800–1820 period, the agreement is good except in Iberian peninsula. For the 1820–1840 period, there is a good agreement except in North Africa and Iberian peninsula. There is also an important discrepancy in NW Europe during the same period. The RMSD was generally below 0.4°C with some time slices about 0.5°C. Both mean curves are in agreement (Fig. 3, lower panel), except at around 1790–1800, 1880–1900, and 1940–1950. These results validate most of the reconstruction as well in the spatial distribution than in the mean trend.

**Table 1:** Comparison of the reconstructions with previous works.

Init date	End date	RSMD Casty	RMSD Luterbacher
1669	1680		0.90
1680	1700		0.72
1700	1720		0.31
1720	1740		0.78
1740	1760		0.69
1760*	1780	0.51	0.53
1780	1800	0.49	0.57
1800	1820	0.31	0.55
1820	1840	0.37	0.54
1840	1860	0.32	0.67
1860	1880	0.54	0.75
1880	1900	0.43	0.96
1900	1920	0.32	0.45
1920	1940	0.43	0.61
1940	1960	0.26	0.50
1960	1980	0.26	0.23
1980	2000	0.58	0.40
correlation	Before 1850	0.00	−0.15
correlation	After 1850	0.36	0.22

*The time-slice 1760–1780 for Casty starts in fact in 1766.

Square-root of mean squared discrepancies (RMSD) between the 20-yr mean maps of this paper and the 20-yr mean maps of [19] and between the 20-yr mean maps of this paper and the 20-yr mean maps of [20]. The last two rows present the correlation between our mean time-series and those of Casty and Luterbacher on respectively the common years before 1850 and after.

We also compared our reconstruction with the LUTER dataset [20] even if some proxies are shared. Unlike LUTER, we used low resolution data for the low frequency domain, reducing the potential redundancy. Table 1 shows that the concordance with LUTER is optimal for the 20^th^ century, but is generally less robust than with CASTY dataset. This concordance degrades before 1900 and remains lower than with CASTY dataset. [32] have previously noted such discrepancies between reconstructions from biological proxies and instrumental data prior the 20^th^ century. This reduced agreement may be attributed to the lower quality of the ancient instrumental observations. Our reconstructions seem less sensitive to these divergences.

**Figure 4:**
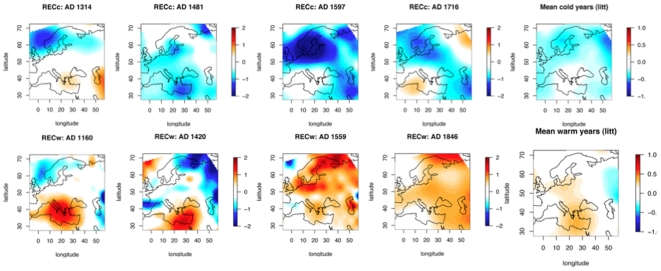
Extreme warm and cold years. The upper panel represents April to September temperature anomaly reconstructions for four years known for the literature to be cold, and the middle panel represents known warm years. The lower maps are averaged maps on all the selected years pointed out in Table 2.

Our reconstructions are generally consistent with the historical literature. Years documented in the literature as very warm or very cold are listed in Table 2 [33], [34], [35], [20]. We found that 75% of the years known as cold are reconstructed as cold (i.e. blue is dominant in the maps); a few are reported in Fig. 4. For the warm years, 65% of the years are reconstructed as warmer than the reference period. In the average map calculated on the 23 warm years of Table 2, we found that most of Europe, but not Eastern Europe, was indeed warm. Temperature at individual grid-points ranges from −0.4 to 0.5°C. Therefore, agreement between our reconstructions and reported literature was poorer and less homogeneous in warm years than cold years.

**Table 2:** List of the cold and warm springs and/or summers.

Century	Cold years	Warm years
12th		1160
13th		1236
14th	1314 1340	
15th	1453 1481	1415 1420 1434 1473
16th	1527 1529 1542 1570 1579 1587 1597 1600	1523 1540 1559
17th	1601 1608 1614 1621 1627 1628 1641 1649 1650 1675 1692 1699	1603 1611 1661 1676 1680 1685 1686
18th	1701 1709 1714 1716 1770 1799	1728 1774 1781 1783 1788
19th	1805 1812 1816 1817 1838 1843 1845 1884	1811 1846
20th	1912	Not used

Sources are literature (Pfister, 1993; Le Roy Ladurie, 1967, 2007; Luterbacher et al, 2004). The years which disagree with our reconstruction are underlined.

### 5. Comparison with long local reconstructions

**Figure 5:**
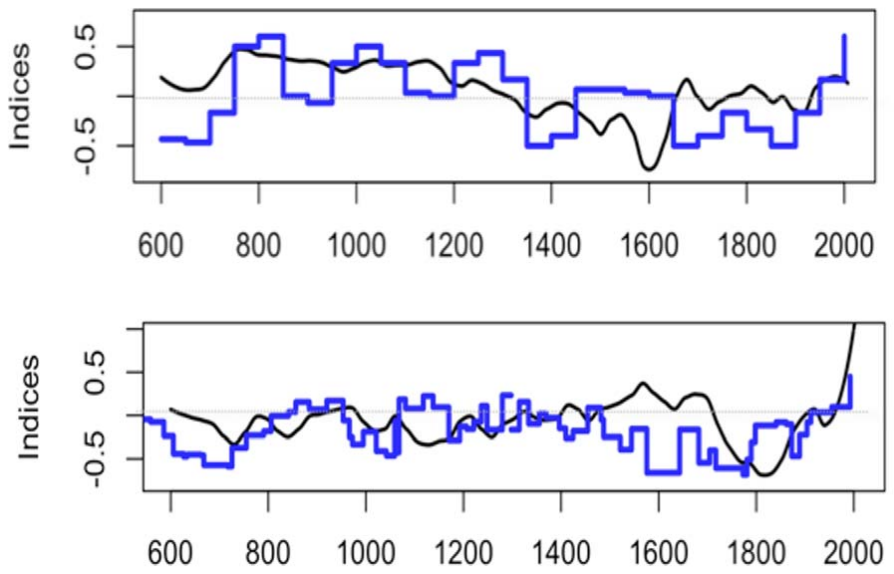
Comparison of the reconstructions and glacier curves. In black, the smoothed April-September temperature of the [7.5°E, 47.5°N] and [2.5°E, 62.5°N] grid-points and in blue, the advance and retreat glacier curve of respectively Aletsch glacier and group of Scandinavian glaciers [22], [23]. The temperature curves are in °C (anomalies) and the glacier curves in dimensionless units.

Fig. 5 compares curves of glacier advances and retreats with temperature reconstructions. Because of the complexity of the climatic signal recorded by these proxies (see section 3) only long term variations of glacier curves are expected to match our reconstructions. The Aletsch curve shows a long retreat phase from 750 to 1350, that corresponds to high temperatures. Between 1350 and 1900, there is a long advance phase (negative index) that corresponds to the LIA. Prior to 750, our temperature reconstruction appears to lack reliability as the Aletsch Glacier was at its maximum and temperatures were higher than the reference. For the South-Scandinavian curve, the period of 800–1450 was marked by glacier retreats (positive index), followed by an advance (negative index) from 1450 to 1900. The main disagreement with our reconstruction was found prior to 700 and from 1500 to 1750. This suggests that the reconstruction between 750 and 1200 is acceptable, although it has a low number of high resolution proxies (<40). The pre-750 period should be taken with much more caution, as less than 20 high resolution proxy series were available (Fig. 1B).

### 6. Volcanic activity

**Figure 6:**
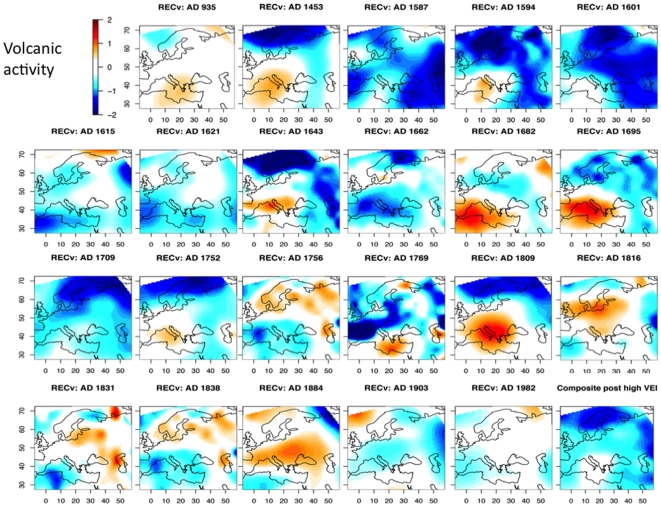
Maps of April to September temperature anomaly reconstructions for the coldest reconstructed year among the three years starting at a volcanic eruption event. The most explosive eruptions have been analysed, for example: 1991 (Pinatubo, Philippines), 1912 (Novarupta, Alaska), 1902 (Santa Maria, Guatemala), 1883 (Krakatoa, Indonesia), 1815 (Tambora, Indonesia), 1783 (Laki, Iceland), 1600 (Huaynaputina, Peru), 1452–1453 (Kuwae, Vanatu), and 934 (Laki, Iceland); but also the events with a large volcanic explosive index (VEI) such as reported by [29], [30]. The lower right map is a composite map calculated as an average of the 22 other maps.

In Fig. 6 shows years following a volcanic eruption: either the year of the eruption, the year after the eruption or two years after the eruption, depending on which year was the coldest in our reconstruction. The coldest year was the year after the Huaynaputina (Peru) eruption in 1600. The year after the Laki (Iceland) eruption in 934 was less cold. A few eruptions give a heterogeneous pattern in 1682, 1695, 1756, 1809, 1816, and 1884, but generally northern Europe was very cold. The western Mediterranean region was the coldest in 1615, 1621, 1662, 1756, 1769, 1838 and 1884. Average for all these years is also displayed in Fig. 6. The range of the grid-points is reconstructed [−1.5, −0.2°C]. This composite map demonstrates a clear negative effect and can be considered as a picture of volcanic forcing in Europe.

### 7. Solar activity

**Figure 7:**
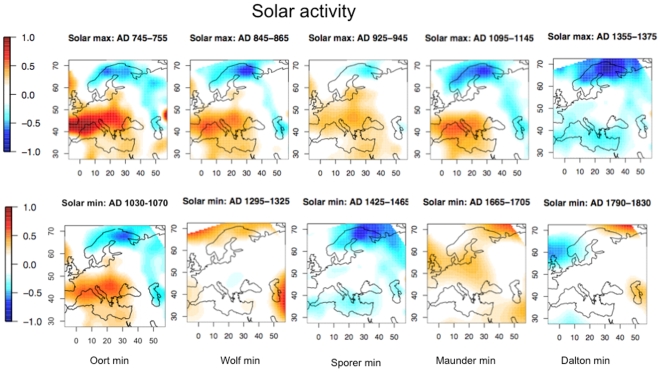
Maps of April to September temperature anomaly reconstructions averaged for time-slices corresponding to high and low solar activity.

To study the effects of the solar activity on the temperatures, Fig. 7 (upper panel) report the mean temperature anomaly maps for periods with high solar activity. These periods of high solar activity are warm in most of Europe with the exception of the last period at the end of the MWP (1355–1375). An explanation for this trend could be human action, as anthropogenic deforestation was intense during this period. Modeling experiments pointed out that possibility that this factor can be responsible for a summer cooling of 0.1 to 0.5°C [12]. After this period, solar activity was generally low until the 20^th^ century. During the 1950–2008 period, solar activity, was higher than that ever recorded during the whole Holocene [31]. It was also dominated by greenhouse gas forcing, which produced a more global warming than in previous periods.

Fig. 7 (lower panel) presents the maps corresponding to low solar activity (Oort, Wolf, Sporer, Maunder and Dalton minima). During these periods, only the Sporer minimum period was clearly cold, while the Oort minimum period was cold in northern and eastern Europe and the Dalton minimum period was cold to the west. This is in disagreement with findings by [36] of a clear fingerprint of the Oort, Sporer and Maunder minima from reconstructions in the Alps. For the Maunder minimum period, LUTER does not find a clear temperature depression either, at least in mean. Hence, it is difficult to conclude that solar forcing has always been the dominant forcing in the spring/summer regimes of Europe.

### 8. Analysis of the spatial patterns of the reconstructions

**Figure 8:**
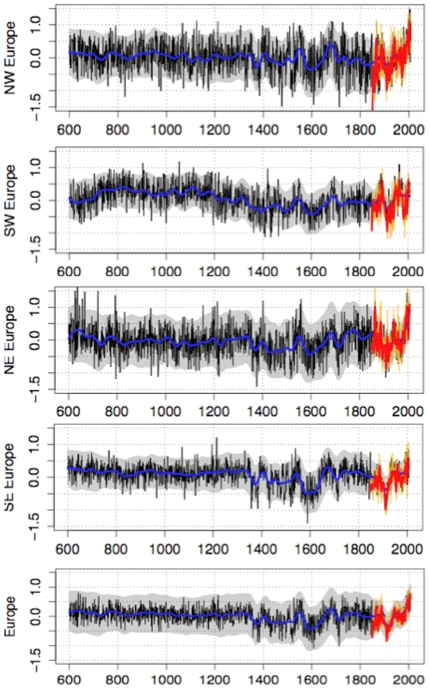
Mean April to September temperature anomalies averaged on various parts of Europe. The black lines represent the reconstructions, the smoothed smoothed confidence interval at 95% are displayed in grey, the blue line is the smoothed reconstruction, orange and red lines are respectively raw and smoothed observations. (a) Eur_SW, Europe south of 45°N and west of 20°E, (b) Eur_SE, Europe south of 45°N and east of 20°E, (c) Eur_NW, Europe north of 45°N and west of 20°E, (d) Eur_SE, Europe south of 45°N and east of 20°E, (e) Eur_mean, whole of Europe. The gridpoints for which RE<0 were excluded from the average.

Fig. 8 shows the mean reconstruction for each quarter of the continent divided by the 20°E meridian and the 45°N parallel. Southwest Europe displays a strong MWP from 750 to 1200 that is comparable to the recent warming. From 1200 to 1900, the mean climate was colder with frequently very cold years. Southeastern Europe temperature variations were more constant across the entire Middle Ages with a mean temperature anomaly of about +0.2°C. After 1300, the temperature variability increased with maximum cooling around 1600 and 1900. Recent warming pattern is very clear. The warmest year of the last decade was 2005, which has been exceeded only two times in the last 1400 years (1195, 1214).

Northwest Europe was generally warm before 1400. Thereafter, temperature declined and became more variable with a strong minimum between about 1600 and 1900. The last decade was clearly over +1°C, with 1995, 1997, 2002, 2004, 2005 and 2007 (four years on the last eight years) surpassing this threshold. The warmest year of the whole period was 2005 with a temperature anomaly of +1.5°C. Northeast Europe had a relatively short warming period before 750, and a cold period from the end of 14^th^ century to the mid 18^th^ century, as well as at the end of 19^th^ century. The recent period passes the threshold of 1.4°C in 1995 that had been reached previously by only four years (629, 636, 637, 698), but this period is far less reliable (see section 5).

The temperature averaged over Europe shows a MPW period ending at about 1350. This period was relatively stable after which the variability increased. Two cold periods can be clearly identified at around 1600 and at the end of the 19^th^ century. Two other cold periods are less pronounced and occurred at around 1700 and 1800. The overall warmest year in Europe was 1997 (+0.9°C) and this anomaly was reached only twice during the last 1408 years (703 and 1697).

**Figure 9:**
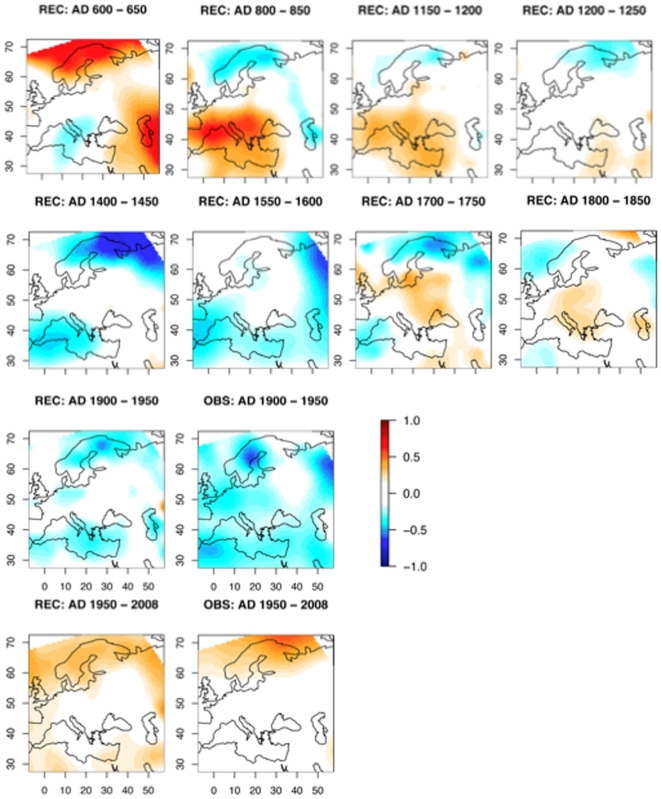
Map of April to September temperature anomalies averaged on selected 50-years periods. REC  =  reconstructions, OBS  =  observations.

**Figure 10:**
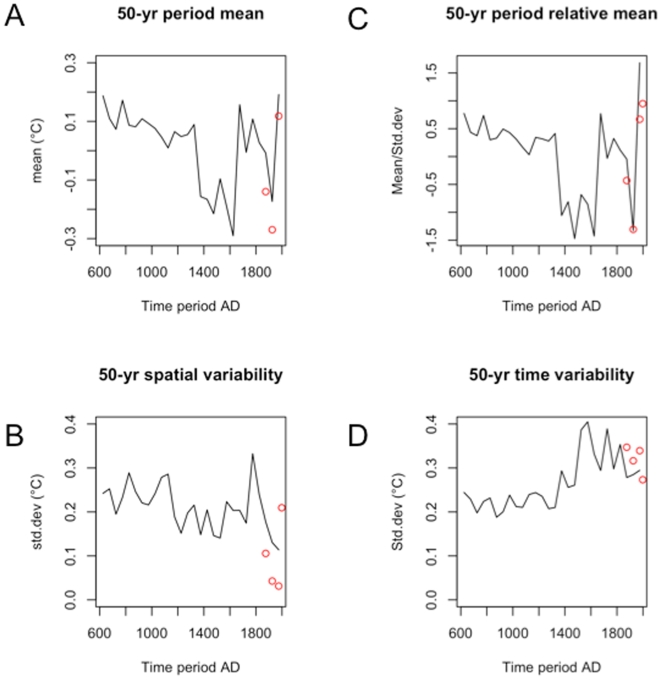
Comparison of means and variances. (A) Sliding spatial mean and (B) variance calculated on 50-yr maps (we have first calculated 50-yr maps and then averages and variances of these maps). Panel (C) represents the ratio of curves (A) and (B). Panel D represents the standard deviation of the European mean temperature by 50-years intervals. Black lines represent the reconstructions and red circles represent the observations.

The 600–1350 period was rather warm, but it was never homogeneous at the continental scale (Fig. 9). By 800, the warm pole shifted from north-east toward south-west. After 1250, a cooling period appeared again in the south-west and extended progressively toward Europe by 1400. The cooling persisted (with some fluctuations) until the first half of the 20^th^ century. The period of 1950–2007 is distinguished from previous warm periods as it was more uniform. The present reconstruction enhances this finding in comparison to direct observations. The sliding average of 50-yr maps, reported in Fig. 10, shows a mean temperature that is similar during the Middle Ages to the last 50-yr period (panel A). However, spatial variances calculated on the same maps show that the 1950–2007 map has a smaller variance than the medieval maps (panel B). This gives evidence that the mean trend and the decrease in spatial variance both being important indicators of global warming during the last decades. The ratio mean on the standard deviation curve was at its minimum in the 20^th^ century (panel C). Red circles in this figure show that the observations are consistent with reconstructions. This double signal of an increased trend and decreased variance may be related to the more global scope of the forcing in action today.

Another important point relates to the time variability of reconstructions (panel D) that is, represented by the temporal standard deviation of the mean European temperature by intervals of 50 years (temporal SD). An excessive decrease of this variability could be considered a signature of insufficient sampling in which the proxy number is small. However, the standard deviation is about 0.3°C after 1500 and about 0.2°C prior to this time. It remains constant before 1200, even with proxy numbers reduced to less than 40. This supports that our method is robust regardless of proxy numbers, unlike methods such as nested regression (see section S1.2 of appendix S1) and is further evidence for the quality of our reconstructions.

**Figure 11:**
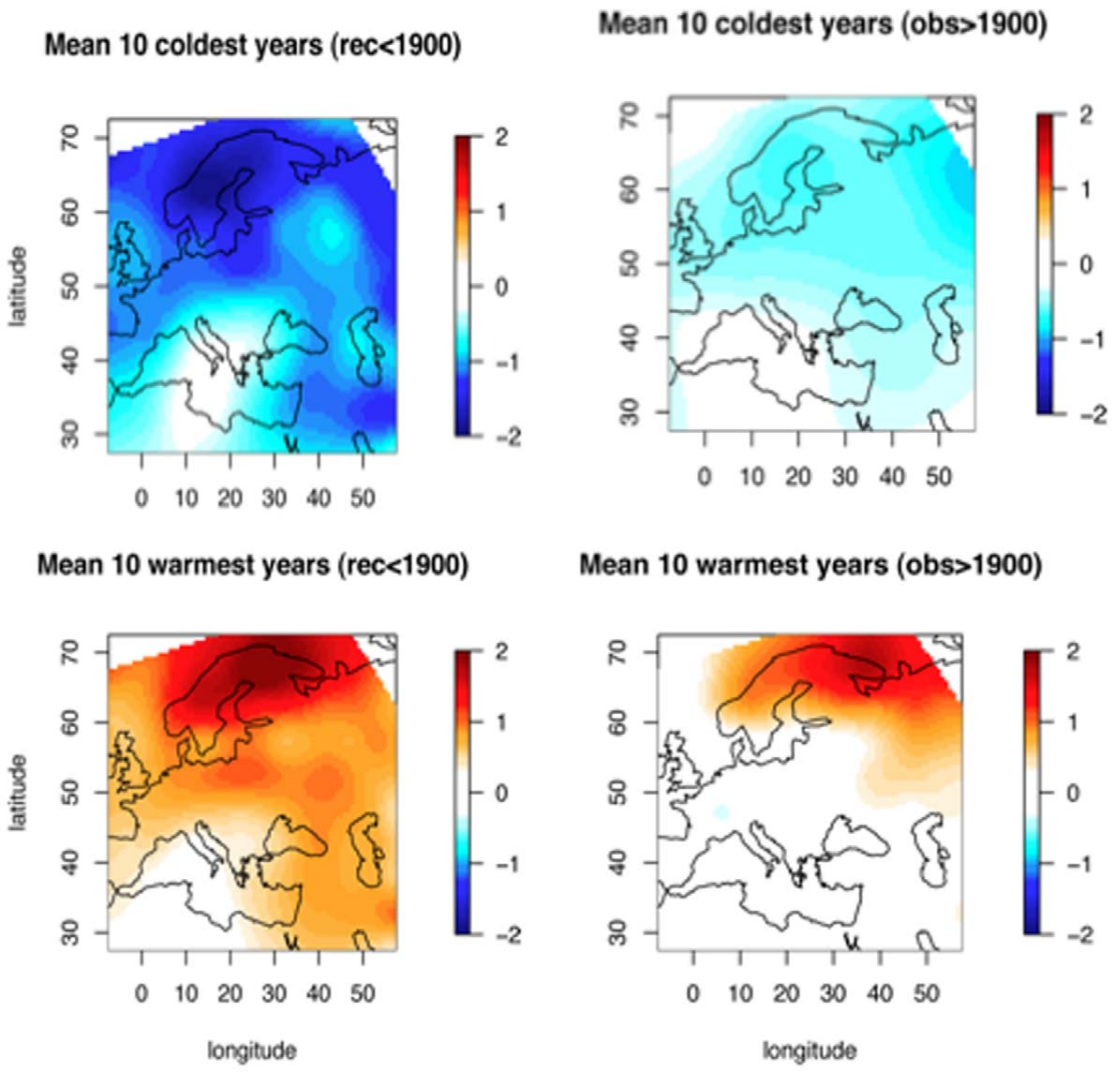
Comparison of extreme years before and after 1900. The upper panel displays the averaged map on the 10 coldest years before 1900 and after; the lower panel displays the 10 warmest years. All values are April to September temperature anomalies.

As depicted in Fig. 11, we analyzed the 10 coldest and 10 warmest years before 1900, as reconstructed by our method. We compared these warm years with 10 extreme observations after 1900. The average map of the 10 coldest years is colder before 1900 than after. This is a consequence of the LIA being over at the beginning of the 20^th^ century. Both maps show a gradient from the north-east (colder) to the south-west (warmer). This spatial structure, linked to the trajectories of the low pressure zones, seems to be the “normal” atmospheric situation of cold spring/summers during the 20^th^ century. As the reconstruction method proceeds by analogy, it was expected that the spatial structure would be similar in reconstructions and in observations. Indeed, it is true for the warm years, but the pattern is less contrasted for the cold years.

### 9. Implications of the methodology

The main advantage of the methodology used, according to other methods is that the long-term variations of climate are reconstructed independently of the short-term variability. It is amplified by the fact that the reconstruction technique is not a regression-based but a similarity-based technique. When a calibration is necessary, the reconstructed variance is necessarily reduced by a factor R^2^, i.e. by a factor two for most of the published reconstructions and even more when the number of proxies much decreases in older times (see an example with [37]). It is why it has been proposed to rescale the variance of the reconstruction [36], [38]. It is not necessarily a solution as it introduces uncontrollable results in the extrapolation [39]. The similarity-based methods as the modern analog technique are, in principle (i.e. when the analogs have a true physical sense), able to reproduce the variance of the reference period, but, at the contrary, they are not able to extrapolate outside the variability of the reference period. Both characteristics of our method (independence of long and short term variations and absence of variance reduction) are particularly pertinent to discuss as well the long term variations of temperature as their extreme values.

### 10. Concluding remarks

This work examined the intensity of recent warming and its spatial structure. To compare recent warming patterns with previous centuries, it was necessary to carefully check the quality of the analog approach, particularly in its ability to provide unbiased reconstructions in terms of mean and variance. Unlike reconstruction approaches based on regressions, we have shown that the variance of analog based reconstructions is not significantly affected by the number of involved proxies. This feature makes it possible to propose several important conclusions.

The first conclusions concern the natural forcings of April to September temperature variations in Europe. We have shown that the few years after a volcanic eruption are generally much colder than the reference period. On average and depending of the location, temperatures vary from −1.5°C to −0.2°C. This confirms the significant cooling effect of volcanic activity. The effect of solar activity is not so clear. High activity periods seem to generally correlate with high temperature. However, among low activity periods, only the Sporer minimum (1425–1465) was very cold, the Oort minimum (1030–1070) was cold in northern and eastern Europe, and the Dalton minimum (1790–1830) was cold in the west. These results are not necessarily in agreement with previous published results. However, as previous papers are not necessarily coherent, the only possible conclusion is that solar forcing has not always been the major forcing in the past everywhere.

Our reconstruction agrees with what is known about the MCA and the LIA. The 600–1350 period was rather warm, but it was never homogeneous at the continental scale. By 800, we reconstructed a warm pole in the south-west. After 1250, a cooling appeared over the European south-west and extended progressively to entire continent by 1400. The cooling remained with some oscillations until the first half of the 20^th^ century. The 1950–2007 period appears unique based on previous warming periods because it was quasi-uniform. The results support the fact that the last decade appears warmer than ever in the last decade and that we are then living a climate change in Europe never known during that period.

## Methods

The method we used is outlined in Fig. 2 and further details can be found in the appendix S1. We briefly present main milestones numbered between brackets. In the proxy space, 32 proxies are available (see section S2.1 of the appendix S1): (1) the first 11 principal components (PC) of the 95 tree-ring proxies (44% of the total variance) (Fig. S2 shows the first three PC), (2) 10 historical series, (3) seven ice-cores series and (4) four PC of the temperature series reconstructed from pollen data (Fig. S3). These 32 series form a matrix with gaps that are represented by blank rectangles and are sorted from highest resolution (yellow) to lowest resolution (green) (1). By using the analog method (AM), the gaps are infilled (result of step AM, section S1 of appendix S1) (2). In section S1.2 of appendix S1 and Table S2, we have shown that the AM properly estimates missing data and extrapolates series having a common signal. The series were decomposed (SD) into three frequency bands by using three complementary filters: high frequency (HF), medium frequency (MF), low frequency (LF) (3). The matrix of April to September mean temperatures (157 years, 125 grid-points) (4) is also in-filled in parallel using the same AM (5) (see examples in Fig. S4). To reduce the noise and decrease the size of the temperature matrix, this in-filled matrix is transformed into 10 PC explaining 68% of the total variance (6). This matrix is decomposed into three complementary bands using the same three filters HF, MF and LF filters described before (7). Their frequency properties are shown in Table S3.

Reconstruction of the 10 PC in each frequency band is also based on the analogs defined respectively on the HF, MF and LF proxy series, respectively (Fig. S7, Table S4). We excluded those proxy series in the frequency band that had biased resolution. Pollen-based temperature series have no variance in HF and MF and they were therefore excluded in these bands. Tree-ring variance is often considered as biased or under-estimated in the LF band [5]. The frequency characteristics of each proxy series are illustrated in Fig. S5. The AM is applied to extrapolate the temperature series back to 600 for each frequency band.

The independent verification of the method was done by randomly excluding an 11-year block from 1851–2007 (see section S2.2. of appendix S1 and Fig. S6 for more explanations). We estimate each temperature PC for each year and each band (8). The three frequency bands are recombined by summation (SR) and provide estimates of the 10 PC (9). PC are transformed into 125 temperature estimates by back-transformation (PC^−1^) (10). Stages (8) to (10) are repeated 100 times with different 11-year blocks taken randomly. Temperature estimates from the year falling at the mid-point of the 100 blocks are considered for an independent validation. Comparisons (11) of these 100 estimates with the observed temperature from the included years provide calibration statistics (see section S2.3 of appendix S1, Table S5, Fig. S8, Fig. S9, Fig. S10). The resulting statistics are then summarized into median and 95^th^-percentile confidence intervals. We applied standard statistics in dendroclimatology that are presented in section S1.2 of appendix S1. The contribution of the main proxy series to the reconstructions is given in section S2.4 of appendix S1, Table S6 and Fig. S11.

## Supporting Information

Appendix S1 Details of the method.
(0.27 MB PDF)

Table S1 Details and references of the proxies used.
(0.05 MB XLS)

Table S2 Verification statistics for the estimation of 101 proxy data using the best analogues taken from the 1575–1744 reference period (the initial number of tree-ring series was 95, that of the historical series was 11, and that of the ice series was 8; 13 series were removed because insufficient number of data within the reference period) R is the correlation between estimates and observations on the reference period, Rp is the verification correlation on the data outside the reference period, RE is the reduction of error and CE the coefficient of efficiency (see text). A cross (+) indicates a confidence level of 75%, a star (*) 90%, two stars (**) 95% and three stars (***) 99%. These confidence levels are determined by Monte-Carlo simulation based 100 red noise series generated for each proxy series. Each random series follows an autoregressive order 1 process where the error term follows a Gaussian law and the order 1 autoregression term is that of the corresponding proxy series.
(0.04 MB XLS)

Table S3 Variance of the 10 temperature principal components: “Total” line concerns the complete spectra and the three other ones the low-pass, middle-pass and high-pass bands. The three bands are delimited by the cut-off frequencies of 0.05 and 0.3 cycles/year.
(0.02 MB XLS)

Table S4 Calibration and verification statistics for each frequency band of the 10 temperature PCs. R is the correlation between observations and estimates on reference data, Rp the corresponding correlation on independent data. R2 is the squared R. RMSEP is the root square of the mean error calculated on the independent data (h-block Jack-knife method, see text). RE is the reduction of error and CE the coefficient of efficiency, both calculated on the independent data.
(0.03 MB XLS)

Table S5 Calibration and verification statistics for temperature series in each grid-point. R is the correlation between observations and estimates on reference data, R2 is the squared R. RE is the reduction of error calculated on the independent data. MPI95 is the 95% mean prediction interval, representing the one-side error bar for the reconstructions at the 95% level.
(0.04 MB XLS)

Table S6 Correlations between each proxy and the 125 reconstructed temperature series (one for each grid-point): we have represented the minimum, mean and maximum of the 125 correlations and sorted them according to the maximum value.
(0.03 MB XLS)

Figure S1 A few tree-ring series. Observations are displayed by red dots and estimates by black lines (estimates done by using the best analog method applied exclusively on the tree-ring series).
(3.00 MB TIF)

Figure S2 The first three principal components of the tree-ring series.
(3.00 MB TIF)

Figure S3 The first five principal components of the gridded annual temperature reconstructed from pollen data by [15].
(0.56 MB TIF)

Figure S4 Mean April-September temperature in grid-points (27.5°N, 57.5°E) (A) (37.5°N, 42.5°E) (B) and averaged on the whole continent (125 series on 10°E to 60°W and 25°N to 75°N) (C).
(1.58 MB TIF)

Figure S5 Scheme of the spectral characteristics of the proxies and temperature series: double red arrows indicate the frequency range of each proxy type in number of cycles per year.
(1.66 MB TIF)

Figure S6 Proportion of variance in common between years separated by various lags from 0 to 30 (squared autocorrelation function). 0-lag represents the standardised variance, i.e. 1. The horizontal line represents the 50% variance.
(0.63 MB TIF)

Figure S7 Estimates versus observations for the first PC of the HADCRUT3 April to September temperature series in the three frequency bands. Blue dots are data used for calibration and red dots data used for the h-block Jack-knife (leave 1-block out) verification. (a) in the low frequency domain, (b) in the middle frequency domain, (c) in the high frequency domain, (d) recombined estimated series (black) and observations (red) in function of time; the green line is the tendency.
(0.92 MB TIF)

Figure S8 April to September temperature anomalies averaged on Europe: observation (red) versus estimates (black); (a) in the low frequency domain, (b) in the middle frequency domain, (c) in the high frequency domain.
(1.73 MB TIF)

Figure S9 Spatial distribution of R, R2, RE and MPI95 (see text).
(1.28 MB TIF)

Figure S10 Comparison, on the reference period 1850–2007, of the reconstructed and observed April to September temperature anomalies in four quarters of Europe and for the whole continent (the quarters are divided by the 45°N parallel and the 20°E meridian). In orange, the observations; in black the reconstruction with its shaded 95%-confidence interval; in blue the trend of the reconstruction and in red the trend of the observations.
(1.56 MB TIF)

Figure S11 Distribution of the correlations between each proxy and the reconstructed temperature series. The proxies were sorted by decreasing order of maximum correlation (in absolute value) and only the proxies with significant correlations are presented. Two scales of correlations are used: for the first seven maps scale from −0.5 to 0.5 and for the others series, scale from −0.3 to 0.3 applies.
(2.26 MB TIF)
