# Supplementary material for: Growing Season Temperatures in Europe and Climate Forcings Over the Past 1400 Years
Source: PLoS One. 2010 Apr 1;5(4):e9972. doi: 10.1371/journal.pone.0009972 (PMC2848609; doi:10.1371/journal.pone.0009972)
Supplement: Appendix S1 — Details of the method. (0.27 MB PDF) [file pone.0009972.s001.pdf]

Appendix S1: Details on the method

S1. The analog method

The multi-proxy reconstruction of climate is a difficult problem as the proxy series have different lengths and some of them, especially the historical document-based series, have gaps. When the problem is limited to the heterogeneity in series length, one may use the nested regression, i.e. a regression is calibrated for each different subset of available predictors. This heavy procedure often leads to reconstructions with a variance decreasing with the number of really included predictors [1]. An alternative method, which is able to deal with missing data anywhere in the time-series, is the regularized expectation maximization (REGEM) algorithm [2]. It imputes missing values in a manner that makes optimal use of spatial and temporal information in the dataset. [3] have proposed another method, the modern analog method, which has proved his efficiency in other domains of paleoclimatology [4,5].

S1.1. Description of the method

It consists in infilling the missing data in the proxy matrix on the basis of the similarity between the annual vectors (and not on the basis of the correlation between variables, as most of the methods do). We define a distance index d_{ik}^2 between year i (for which we need an estimate) and year k (used to fill the gaps in year i). This distance measures how close these two vectors of data are:

$$d_{ik}^2 = m_{ik}^{-2} \sum_{j=1}^{m} \left(\frac{(x_{ij} - x_{kj})^2}{(x_{Mj} - x_{mj})^2} \delta_{ik}^j \right) \tag{1}$$

where x_{ij} represents the value of variable j in year i, x_{kj} the value of variable j in year k, $(x_{Mj} - x_{mj})$ is the amplitude (difference between maximum M and minimum m, both being determined on the interannual variability of each proxy) of variable j calculated using all data available. δ_{ik}^j is an index equal to 1 if a value is available for variable j in year i as well k, and 0 otherwise. The sum is computed for the m_{ik} components of the m-vector where data are available for both years k and i, *i.e.*

$$m_{ik} = \sum_{j=1}^{m} \delta_{ik}^j \tag{2}$$

Equation (1) has the squared m_{ik} at the denominator to make all the distances comparable, *i.e.* independent of the number of proxies available. We use the best analog available for year i, *i.e.* the vector satisfying the conditions of having the smallest distance and data available for variable j. This is a difference with the method used by [3] who used a mean of all the analogs with a distance <0.5, with the risk to smooth the reconstructed variance. The choice of distance metric has an influence on the results. In pollen analysis, the chord distance where the pollen frequencies are transformed into square roots is often preferred. It does not apply for our data. We have chosen the simplest distance, the Euclidian distance, because our trials have shown that it performs as well as other methods.

S1.2. Validation of the analog method

The quality of fit by the analogs is evaluated with the correlation between observations and estimates on the common period. A good correlation proves that the hypothesis justifying the use of analogs is satisfied, *i.e.* that the notion of analog has a meaning. Nevertheless, these correlations can be overestimated because of time autocorrelation in the proxy series. If, systematically, the best analog is found just before or after the missing data, we have not guarantee that for years much distant, the estimates remain acceptable. We have now to test that a meaningful analog actually exists irrespective of the temporal location of the missing data. Ideally, this could be done by testing the climate reconstructions all along the time periods where they are obtained, but the shortness of the instrumental climate data prevents it. An alternative way is to use the proxy series themselves for the validation, assuming that the proxies are the climate that must be reconstructed. This hypothesis is not too strong as the proxies have been chosen because they are related to climate. If we are able to reconstruct the proxies of any year of the considered period from a limited time period considered as the calibration or the reference period – as it is done with the instrumental periods -, we may consider our method as robust.

Fig. 1B shows the number of proxies available for each year. The maximum of data is found between A.D. 1500 and 1900. The reference period, for this test, is taken within that interval to maximise the number of proxies available. If we impose a minimum of 100 proxies, the period reduces to A.D. 1575-1744, i.e. 170 observations, a number close to the length of the instrumental series. For a given proxy, each year, where data are available, is estimated by the best analog taken from the A.D. 1575-1744 reference period. The validation is done by using four statistics:

- R, the correlation between observations and estimates on the reference period (1575-1744, 170 observations);
- Rp, the correlation between observations and estimates on the data outside reference period; the number of data available depends on the series;
- RE, the Reduction of Error, is the proportion of variance explained by the analogs, calculated relative to the reference mean;
- CE, the coefficient of efficiency, is also proportion of variance explained by the analogs, calculated relative to the mean of the data not belonging to the reference period.

The RE statistic ranges from minus infinity to one. If this quantity is greater than zero, the reconstruction has greater skill than that obtained by simply using the mean of the reference data as analog [6]. The CE statistic is often used in the tree-ring literature [7]. It is useful when the instrumental data are not stationary.

The test is applied to the 101 proxy series with a minimum of 50 observations in the reference period. To calculate confidence intervals for these statistics, a Monte-Carlo technique is used. A set of 101 random time-series taken from Gaussian white noises are generated and transformed into red noise with a first autocorrelation equal to that of each proxy series. The estimates are obtained by the analogs method. This is repeated 100 times (with different random series) and the four statistics, earlier mentioned, are calculated and summarized by the 50th, 75th, 90th, 95th and 99th percentiles. When a statistic passes one of these levels, it is considered as significant at the corresponding level. Table S2 shows (1) that, even if the correlations R are high, they are not necessarily better than those obtained on random series (31% only are significant at the 90% level) and that this parameter is not reliable because of the autocorrelation of the series; (2) that the large majority (around 60%) of Rp, RE and CE are significant at the 75% level and 50% at the 90% level, and then that the

analogs are meaningful to reconstruct past climates; (3) that a non negligible part of the proxies (40%) are not well estimated, which means that they do not contain a sufficiently large portion of climatic variance.

The fact that some series are poorly reconstructed means either that they do not contain any climatic signal or that they the climatic signal recorded is more complex. It is impossible to choose between both alternatives. If the second one prevails, there is an important loss to eliminate these series. If the first one prevails, it is not certain that they will have a strong effect on the whole reconstructions. We decided to keep them.

Analogue method performs well if two similar years are driven by similar climate forcing. This test proves that it is the case. The method has another interesting characteristic as compared with the regression-based methods: the correlations between estimated series are not better than those of the observed series as the estimation process is not based on the similarity between the variables but between the years. The method is then conservative for the observed spatial variability. It is demonstrated by [8].

Recently, it has been pointed out that the spatial autocorrelation had the consequence to over-estimate the fit when it is applied to spatial data [9]. This is true also for temporal analogs when proxy-series are red noise series. The problem has been avoided here by separating the target year and the analog by a minimum number of years (see below h-block Jack-knife technique). The analogs selected are necessarily far (at least by 5 years) from the reference set.

S2. The reconstruction methods and results

The proxy series are very heterogeneous as well in their length than their resolution. A three-step method has been designed to homogenise their length and to obtain a matrix covering the maximum period of A.D. 600-2007 before calibrating the climatic signal. The same method is used as well to homogenise the length of the temperature series on the A.D. 1850-2007 period and to extrapolate the temperature series from the proxy series.

S2.1. The pre-processing step

First, missing data of the tree-ring series are infilled using the analog method. This initial 1404x95 matrix has 57% of gaps induced by the length heterogeneity of the series. Previous section showed that the correlation R was a biased indicator of the quality of fit, as it is possible to have correlations up to 0.70 with red noise series. Nevertheless, the large majority (73%) of the correlations calculated between observations and estimates are larger than 0.70, which is a clue of the quality of the fit. This complete the conclusion based on Table S2, that the analog method is relevant for the proxy series.

Fig. S1 shows a few examples of infilled series. The three series called Torne1, Torne2, Torne3 gives us a good opportunity to evaluate the quality of the analog method. Torne2 has no data before A.D. 1400. The extrapolation before that date is of good quality: it reproduces perfectly the low indices of the 10th century observed in Torne1 and Torne3, which is expected by their geographical position. Moreover, these three series have a common period between 1400 and 1947. The mean correlation of the three observed series is 0.66 and this correlation remains of the same magnitude for the estimates (0.53). We conclude that the spatial heterogeneity between the filled series does not vary according to the number of proxies available. It is an important property for the interpretation of the reconstructions.

This step results in a complete matrix of 95 series and 1404 rows between A.D. 600 and 2003. To avoid an over-representation of the tree-ring series according to the other proxies, this matrix is transformed into principal components. We selected the first 11 PC's, which

explain together about 44% of the total variance. The first three PC's are represented in Fig. S2. The first PC is negatively related to mountain species as *Larix decidua* and *Pinus cembra*, which indicates a clear negative relationship with temperature. The second PC is negatively correlated with *Pinus sylvestris*, a cool temperate species also indicating a temperature signal. The third PC is positively correlated with most of the conifer species and PC4 with the deciduous species (mainly oak). The other PC's are more spatially heterogeneous and potentially indicate local variations of temperatures.

The annual temperature derived from pollen data in Europe [10] are available on a grid of 1236 points between 10°W and 50°E and between 35°N and 70°N, by step of 1° longitude and latitude (Fig. 1A). They have also been transformed into principal components. We retain the first five ones explaining 91% of their total variance (Fig. S3). This small number is explained by the large correlations between these gridded variables. PC1 (42% of the variance) indicates a strong warming during the 19-20th centuries. The second one (24% of the variance) indicates a strong cold period between A.D. 1400 and 1900, which could be related to the Little Ice Age (LIA). The other PC's are more variable and cannot be identified easily with clearly known climatic events. We exclude the fourth one (7% of the variance), because it emphasizes a warm medieval period at the same level than the end of the 20th century, but also a warm 17-18th century which does not correspond to our knowledge of this period. We know that the unequal resolution of the various pollen cores used by (Davis et al. 2003) may be responsible of artefacts. Retained components 1, 2, 3 and 5 explain together 84% of the variance.

The historical and ice-core series are bound to the 11 PC's of the tree-ring series and the 4 PC's of the pollen derived annual temperature to form a matrix of 32 series.

The HADCRUT3 temperature dataset contains 156 series spaced by 5° of longitude and latitude, with about the half of missing data. We kept the 125 series containing more than 50 observations. They are represented by colored circles on the maps of Fig. S9. Missing data are infilled using the analogs method. The correlations between observations and estimates range between 0.34 and 0.80. These values are more meaningful than for the tree-ring series as the temperature series are not autocorrelated. Fig. S4A and S4B show the observed and estimated series for respectively the poorest estimated series (57.5°E, 27.5°N) and the best estimated one (42.5°E, 37.5°N). In the latter case, the low quality of fit is mainly due to the heteroscedasticity of the series. This shows that the infilled series are not all of good quality (18 have a squared-R lower than 0.15), but for a continental reconstruction, they are acceptable. The time series averaged over the whole continent (Fig S4C) has a trend close to the global curve found in the last IPCC report [11]. Before 1940, the anomalies are generally negative and, after 1980, they are positive or close to zero.

The 125 time-series are transformed into principal components to reduce the number of variables. We keep 10 PC's explaining about 68% of the total variance.

S2.2. The calibration step using a h-block Jack-knife spectral analog method

The reconstruction of the 10 PC's of the temperature series will also be based on the analogs defined on the 32 proxy series. To manage the differences in resolution, the spectra of each proxy and temperature PC's series are decomposed into three complementary bands (as already done by [12]) and the reconstruction is achieved separately for each frequency band. The proxies which are not reliable or have no significant variance in a given frequency band are not used for the reconstruction of the corresponding band. Fig. S5 shows which series are considered as reliable in each frequency band. For the low frequency band (frequency $f < 0.05$ or period $T > 20$ years), we rely on ice data, historical written documents and pollen data (21 series). For the intermediate component ($0.05<f<0.3$ or $3.3<T<20$ years), we rely on a part of

ice data, tree-ring series and historical written documents (25 series). For the high frequency component (f>0.3 or T<3.3 years), we rely on tree-ring series and historical written documents (21 series). The temperature PC's are assumed to contain all the frequency components.

Complementary band pass filters

The series are filtered with a band pass filter based on Fast Fourier Transform (FFT) algorithm available for the R-software environment (function "fft" of "base" library [13]). To filter out a frequency band, (1) the direct FFT transforms the series in the frequency domain, (2) all the frequencies within the band are set to zero, and (3) the resulting spectrum is back-transformed into the time domain using the inverse FFT. The low-pass filter is based on the frequency limits [0, 0.05], the middle-pass filter, on the limits [0.05, 0.03] and the high-pass filter, on the limits [0.3, 0.5]. By adding the three resulting series, by construction, we find back the original series and the three filters are said complementary. An illustration of the effect of these filters is given in Fig. S8 for the April to September temperature averaged on Europe.

This algorithm implemented to filter the data can work with missing values. Before filtering, the missing data are replaced by the mean of the series, which may attenuate the mean spectrum power, but not distort it. After inverse FFT application, the initial missing cases are restored.

h-block-Jackknife reconstruction in each frequency band

The main property of any time series is the time autocorrelation. An observation is not necessarily independent on its neighbor. With low-pass filtered time-series, this property is strongly exacerbated. The consequence is that the best analog of a given year will often be the previous or the following one in the time sequence, as already seen in section S1.2. When the results are verified on the instrumental period, this fact makes the fit over-estimated. To avoid that over-estimation, we defined, around each observation used for verification, a h-block where potential analogs are excluded. To facilitate the calculations, we defined the length h of the block identical for the three bands, even if it could be lower for the high frequency band than for the low frequency band. It has been defined on the basis of the squared autocorrelation function (Fig. S6) of the low frequency band of the temperatures, which represents the percentage of variance in common between observations separated by various time intervals Δt. This common variance is 98% for Δt=1 years. It falls at 51% for Δt=6 years and 27% for Δt=10 years. So, we set h equal to 5 years, meaning that it is forbidden to take, for a given year, an analog when it has more than 50% of the variance in common with it, knowing that this variance is just due to filtering. A lower h should make the verification statistics overestimated and a higher value should reduce too much the number of potential analogs.

Table S3 show that all the principal components of the temperature series have a variance dominated by the middle frequency band. The first two PCs have also a large variance in the low frequency band, while the higher order PCs have a larger variance in the high frequency band. It means that the climate trend is contained in the first PCs and that it is particular important to reconstruct them as well as possible.

The reconstruction method is also based on the analogs (section S1.1), but we use here a Jack-knife scheme to check independently the quality of fit and to calculate the error bars of the reconstruction. The Jack-knife scheme is based on N (here set to 100) replications of the reconstruction. For each replication, a year is randomly taken in the time-interval where instrumental data are available. This year is used for an independent verification. A safety 5-

years block is defined around it and the analog method is applied to all the observations, excluding any analog from the safety block. The randomly taken observation is used for the independent verification statistics and the other ones are used for estimating the calibration statistics. It is specified in next section.

This is done for each frequency band. It is illustrated for the first PC in Fig. S7. We use the same test observations for each of the three bands. Then it is possible to sum the three bands replication by replication and to have the same independent verification dataset. At that stage, we have N recombined temperature PC's series which are themselves back-transformed into 125 temperature series. These have been compared together to provide a median reconstruction with a confidence interval and with the observations to provide calibration and verification statistics. As previously said, Fig. S8 shows the mean temperature of Europe, as observed and estimated in each frequency band.

Error bars and verification statistics

The Jack-knife method gives access to confidence intervals as well for the reconstructions than for the verification statistics. The reconstruction skill and its robustness are usually assessed by the Root Mean Squared Error of the calibration data (rmse), the Root Mean Squared Error of Prediction based on the verification data (rmsep), and the previously presented statistics (R, Rp, RE and CE). The RMSE statistic tests the quality of fit on the calibration data, *i.e.* they show if the hypotheses behind the existence of analogs are verified, while the RMSEP tests the prediction capacity of the reconstruction by using independent data. All the observations used for calibration are bounded in one big matrix where the number of raws is equal to 100*(n-1), i.e. the number of replications multipled by the number of reference years (minus the year used for verification). The observations used for validation are the 100 years removed by the Jack-knife procedure. R, rmse and R2 are then based on 100*(n-1) years and Rp, rmsep, RE and CE on 100 years.

Table S4 shows that the low frequency band is well reconstructed. The calibration statistics (R, R^2) are amplified by the autocorrelations. But the verification statistics (using the 5-year safety blocks) are also strongly significant (RE and CE are most often higher than 0.5). The first five PCs, which explain the maximum of variance are also the best estimated. For the two other frequency bands, because of less large autocorrelations, the calibration statistics are lower and the R^2 are close to the RE, which indicates a better stability of the results.

S2.3. Post-processing and final uncertainties

The three frequency bands are recombined and the PC's are back-transformed into temperature variables. The total R varies from 0.33 to 0.73 with a mean of 0.60 and that the total R^2 varies between 0 and 0.48 with a mean of 0.28 (Table S5). Fig. S9 shows the spatial distribution of the whole spectrum R, R^2 and RE statistics. The region from Tunisia to Egypt has a negative RE, which can be explained by the lack of proxy data. There is also low RE at southeast, northwest of the covered region and also in central Europe.

The 95%-prediction intervals are based on the variability of the estimates between the 100 replications. By averaging them on the whole time-period, we obtain 95% mean prediction interval for each grid-point (MPI95). These intervals include as well the uncertainties due to the model specification and due to the sampling. They are relatively large (Table S5), 0.7°C in average. Fig. S9 shows that they reach 1°C in the northern grid-points and falls below 0.6°C in the western part of Europe and in the Mediterranean region (except Spain). This spatial distribution of MPI95 cannot be interpreted as a map of quality of fit, because MPI95 is also dependent on the variance of the observed temperature at the grid-point. The proper parameter is R^2, for the quality of fit, and RE, for the quality of prediction.

They are also acceptable in average: R^2 is 0.32 in mean and RE is 0.16.

Fig. S10 shows the mean reconstruction for each quarter of the continent divided by the 20°E meridian and the 45°N parallel and for the whole continent on the calibration period (1850-2007). Observed and reconstructed temperature fit as well for the high frequencies than for the trends. But, more important, the reconstructed variability is quite similar to the observed one: the standard deviation for observed mean European temperature is 0.37 °C and 0.32 °C for reconstruction. The extremes are then well reconstructed. It is an intrinsic characteristic of the analog method: there is no variance loss in comparison with a regression-based method. The maximum of temperature is +0.9 °C for the observed series and +0.8 °C for the reconstructed series.

S2.4. Influence of proxies on the reconstructions

To verify the influence of each proxy on the final reconstruction, we calculated the correlation coefficients between each proxy (or the PC of the proxy) and the 125 reconstructed temperature series (one for each grid-point). Correlations are presented in Table S6 in decreasing order. Correlation spatial patterns of a few proxies are shown in Fig. S11. The best correlated proxy variables are the pollen PC5 (ppc5) and the Grip and Dye ice series. All these series are of low resolution, demonstrating the importance of the low frequencies in our reconstructions. Pollen PC5 (ppc5) shows an opposition between Mediterranean area and the rest of Europe. This strong pattern is frequently found in reconstructions (Fig. 10). The next low resolution variable is pollen PC2 (ppc2), which is positively correlated with south-west Europe. Optimal correlations with high resolution series (4th and 6th rank) are the annual temperature index derived from written historical documents (Pichard_Tindex_an) and the Rhone grapes harvest series (VEND_LL_Rho). The first tree-ring PC is dcp4 (for dendrochronological series pc4) and was ranked 10th. Among the 11 tree-ring PCs, good correlations were only obtained for the 3rd , 4th, 6th and 7th PCs. These PCs were spatially homogeneous, except for the 4th, which shows an opposition between south-west Europe and the rest of the continent similar to ppc5 (Fig. 3). Most of the series were positively correlated to reconstructions, except for ppc5, the grape harvest dates series and dcp7. However VEND_LL_Rho is surprisingly positively correlated to temperature reconstructions.

These correlation analyses between proxies and temperature reconstructions showed a strong dichotomy between southwest Europe and the rest of the continent, as emphasized by [14]. Correlations are also consistent with expected results from interpretations of the proxies, suggesting that the reconstructions are of high quality.

Cited references (in the text of the SI)

[1] Pauling A, Luterbacher J, Casty C, Wanner H (2006) Five hundred years of gridded high-resolution precipitation reconstructions over europe and the connection to large-scale circulation. Climate Dynamics 26:387-405

[2] Rutherford S, Mann ME, Osborn TJ, Bradley RS, Briffa KR, et al. (2005) Proxy-based northern hemisphere surface temperature reconstructions: Sensitivity to method, predictor network, target season, and target domain. Journal of Climate 18:2308-2329

[3] Guiot J, Nicault A, Rathgeber C, Edouard JL, Guibal E, et al. (2005) Last-millennium summer-temperature variations in western europe based on proxy data. Holocene 15:489-500

[4] Guiot J, Pons A, Debeaulieu JL, Reille M (1989) A 140,000-year continental climate reconstruction from 2 european pollen records. Nature 338:309-313

[5] Devernal A, Guiot J, Turon JL (1993) Late and postglacial paleoenvironments of the gulf of st-lawrence - marine and terrestrial palynological evidence. Geographie Physique Et Quaternaire 47:167-180

[6] Fritts HC (1976) Tree-rings and climate, Vol. Academic Press, New York

[7] Frank D, Esper J (2005) Temperature reconstructions and comparisons with instrumental data from a tree-ring network for the european alps. International Journal of Climatology 25:1437-1454

[8}Corona C, Guiot J, Edouard J-L, Chalié F, Büntgen U, et al. (2008) Millennium-long summer temperature variations in the european alps as reconstructed from tree rings Clim. Past Discuss., 4, 1159-1201

[9] Telford RJ, Birks HJB (2009) Evaluation of transfer functions in spatially structured environments. Quaternary Science Reviews 28:1309-1316

[10] Davis B, Brewer S, Stevenson A, Guiot J (2003) The temperature of europe during the holocene reconstructed from pollen data. Quaternary Science Reviews 22:1701-1716

[11] IPCC (ed) (2007) Climate change 2007: The physical science basis. Contribution of working group i, Vol. Cambridge University Press, Cambridge, UK and New York, NY, USA

[12] Guiot J (1985) The extrapolation of recent climatological series with spectral canonical regression. Journal of Climatology 5:325-335

[13] Becker RA, Chambers JM, Wilks AR (1988) The new s language: A programming environment for data analysis and graphics, Vol. Wadsworth & Brooks/Cole, Pacific Grove, CA

[14] Guiot J (1992) The climate of central canada and southwestern europa reconstructed by contining various types of proxy data : A detailed analysis of the 1810-1820 period. In: Harington CR (ed) The year without a summer? World climate in 1816. Canadian Museum of Nature, Ottawa, p 291-308

[15] Davis B, Brewer S, Stevenson A, Guiot J (2003) The temperature of europe during the holocene reconstructed from pollen data. Quaternary Science Reviews 22:1701-1716

Cited references (in Table S1)

Andersen KK, Ditlevsen PD, Rasmussen SO, Clausen HB, Vinther BM, Johnsen SJ (2006) Retrieving a common accumulation record from Greenland ice cores for the past 1800

years,Retrieving a common accumulation record from Greenland ice cores for the past 1800 years, Journal of Geophysical Research 111, D15106: doi:10.1029/2005JD006765.

Bartholin T, Karlen W (1983) Dendrokronologi i Lapland, Dendrokronologiska Sallskapets Medelanden 4: 3-16.

Becker B, Giertz-Siebenlist V (1970) Ein ûber 1100 jährige mitteleuropaische Tannenchronologie. Flora 159: 310-346.

Billamboz A (1990) Etude dendrochronologique et approche historique d'un village du Jura Comtois. Dendrochronologia 8: 99-117.

Brehme K (1951) Jahrringchronologische und -klimatologische Untersuchungen an Hochgebirgslärchen des Berchtesgadener Landes. Zeitshrift für Weltforstwirtschaft 14: 65-80.

Brown DM (1997) Dendrochronological Dating of Hiberno-Norse Medieval Structures from Waterford. In T Barry, R Cleary, MF Hurley (eds.) Late Viking Age and Medieval Waterford Excavations 1986 - 1992 by M. F. Hurley, O. M. B. Scully and S. W. J. McCutcheon. Waterford Corporation 643-649.

Büntgen U, Esper J, Frank DC, Nicolussi K, Schmidhalter M (2005) A 1052-year tree-ring proxy for Alpine summer temperatures. Climate Dynamics 25, 141-153.

Büntgen U, Frank DC, Nievergelt D, Esper J (2006) Summer temperature variations in the European Alps, A.D. 755-2004. Journal of Climate 19: 5606-5623.

Chbouki N (1992) Spatio-temporal characteristics of drought as inferred from tree-ring data in Morocco. PhD thesis, University of Arizona, Tucson, AZ.

Chuine I, Yiou P, Viovy N, Seguin B, Daux V, Ladurie EL (2004) Historical phenology: Grape ripening as a past climate indicator. Nature 432, 289-290: doi:10.1038/432289a.

Clausen HB, Gundeestrup NS, Johnsen SJ, Bindschadler R, Zwalley J (1988) Glaciological investigations in the Crete area, central Greenland: a search for a new deep-drilling site. Journal of Glaciology 10: 10-15.

Coles S (2001) An introduction to statistical modeling of extreme values. Springer Series in Statistics, Springer-Verlag, London, 208 p.

Cuffey K, Clow G, Alley R, Stuiver M, Waddington E, Saltus R (1995) Large Arctic temperature change at the Wisconsin /Holocene glacial transition. Sciences 270: 455-458.

Dahl-Jensen D, Mosegaard K, Gundestrup N, Clow GD, Johnsen SJ, Hansen AW, Balling N (1998) Past Temperatures Directly from the Greenland Ice Sheet. Science, 282 (5387): 268-271.

Genova Fuster M, Fernandez Cancio A (1999) Tree rings and climate of Pinus nigra ssp. salzamannii in Central Spain.Dendrochronologia 16/17: 75-85.

Genova Fuster M, Fernandez Cancio, A, Creus Novau J (1993) Diez series medias de anillos de crecimiento en los sistemas capetano e iberico. Investigacion Agraria Sistemos y Recursos Forestales 2: 151-72.

Glueck MF, Stockton CW (2001) Reconstruction of the North Atlantic Oscillation, 1429-1983, International Journal of Climatology 21: 1453-1465

Manning SW, Kromer B, Kuniholm PI, Newton, MW (2001) Anatolian Tree Rings and a New Chronology for the East Mediterranean Bronze-Iron Ages, Science 294: 2532-2535.

Guerreau A (1995) Climat et vendanges (XIVe/XIXe siècles): révisions et compléments. Histoire et Mesure 10: 89-147.

Hoffsummer P (1989) L'évolution des toits à deux versants dans le bassin mosan: l'apport de la dendrochronologie (XI-XIXe siècle). Ph.D. dissertation, Université de Liège, Belgium.

Huesken W, Schirmer W (1993) Drei Jahrringchronologien aus den Pragser Dolomiten/Sudtirol. Dendrochronologia 11: 123-137.

Kirchhefer AJ (2001) Reconstruction of summer temperatures from tree-rings of Scots pine (Pinus sylvestris L.) in coastal northern Norway. The Holocene 11(1): 41-52.

Lamb HH (1977) Climate: present, past and future. London, Methuen: 825 pp.

Le Roy Ladurie E (1983) Histoire du climat depuis l'an Mil. Paris: Flammarion, 541 pp.

Le Roy Ladurie E, Baulant M (1981) Grape harvests from the fifteenth through the nineteenth centuries. In Rotberg, R.I. and fifteenth through the nineteenth centuries. In Rotberg, R.I. and NJ: Princeton University Press: 259-269.

Legrand JP (1979) Les fluctuations météorologiques exceptionnelles durant les saisons printanières et estivales depuis le Moyen-Age. La Météorologie VIe série 18, 131-141.

Lindholm M (1996) Reconstruction of past climate from ring-width chronologies of Scots pine (Pinus sylvestris L.) at the northern forest limit in Fennoscandia. University of Joensuu Publications Sciences 40.

Meier N, Rutishauser T, Pfister C, Wanner H, Luterbacher J (2007) Grape harvest dates as a proxy for Swiss April to August temperature reconstructions back to A.D. 1480. Geophysical Research Letters 34, L20705: doi:10.1029/2007GL031381.

Motta R, Nola P (1999) Global change, structure and dynamic at the upper forest limit in the Aleve forest (Varaita Valley, Piedmont, Italy). Global Change and Protected areas, L'Aquila, September 8-13, 1999. Abstracts.

Motta R, Nola P (2001) Growth trends and dynamics in sub-alpine forest stands in the Varaita Valley (Piedmont, Italy) and their relationships with human activities and global change, Journal of Vegetation Science 12, 219-230.

Nola P, Motta R (1996) Una cronologia plurisecolare di Larice (Larix decidua) per l'alta Valmalenco (Sondrio, Italia). Dendrochronologia 31-42.

Pfister C (1993) Historical Weather Indices from Switzerland. IGBP PAGES/World Data Center-A for Paleoclimatology Data Contribution Series # 93-027. NOAA/NGDC Paleoclimatology Program, Boulder CO, USA.

Serre F (1978) The dendroclimatic value of the European larch (Larix decidua Mill.) in the French Alps. Tree-Ring Bulletin 38: 25-34.

Siebenlist Kerner V (1984) Der Aufbau von Jahrringchronologien für zirbelkiefer, Lärche und fichte eines alpinen hochgebirgsstandortes. Dendrochronologia, 2: 9-29

Smith RL (1985) Maximum likelihood estimation in a class of non-regular cases. Biometrika, 72: 67–90.

Tessier L 1981: Contribution dendroclimatique à la connaissance écologique du peuplement forestier des environs des Chalets de l'Orgère (Parc National de la Vanoise). Travaux Scientifiques du Parc National de la Vanoise 11: 29-61.

Touchan R, Xoplaki E, Funkhouser G, Luterbacher J, Hughes MK, Erkan N, Akkemik Ü,

Stephan J (2005) Reconstructions of Spring/Summer Precipitation for the Eastern Mediterranean from Tree-Ring Widths and its Connection to Large-Scale Atmospheric Circulation. Climate Dynamics 25: 75-98.

Van Engelen AFV, Buisman J, Ijnsen F (2001) A Millenium of Weather Winds and Water in the Low Countries in: History and Climate Memories of the Future, Jones et al (eds), Kluwer Academic / Plenum Publishers, 101-123.

Figure Captions

Figure S1. A few tree-ring series. Observations are displayed by red dots and estimates by black lines (estimates done by using the best analog method applied exclusively on the tree-ring series).

Figure S2. The first three principal components of the tree-ring series

Figure S3. The first five principal components of the gridded annual temperature reconstructed from pollen data by [15].

Figure S4. Mean April-September temperature in grid-points (27.5°N, 57.5°E) (A) (37.5°N, 42.5°E) (B) and averaged on the whole continent (125 series on 10°E to 60°W and 25°N to 75°N) (C).

Figure S5. Scheme of the spectral characteristics of the proxies and temperature series: double red arrows indicate the frequency range of each proxy type in number of cycles per year.

Figure S6. Proportion of variance in common between years separated by various lags from 0 to 30 (squared autocorrelation function). 0-lag represents the standardized variance, i.e. 1. The horizontal line represents the 50% variance.

Figure S7. Estimates versus observations for the first PC of the HADCRUT3 April to September temperature series in the three frequency bands. Blue dots are data used for calibration and red dots data used for the h-block Jack-knife (leave 1-block out) verification. (a) in the low frequency domain, (b) in the middle frequency domain, (c) in the high frequency domain, (d) recombined estimated series (black) and observations (red) in function of time; the green line is the tendency.

Figure S8. April to September temperature anomalies averaged on Europe : observation (red) versus estimates (black); (a) in the low frequency domain, (b) in the middle frequency domain, (c) in the high frequency domain.

Figure S9. Spatial distribution of R, R2, RE and MPI95 (see text)

Figure S10. Comparison, on the reference period 1850-2007, of the reconstructed and observed April to September temperature anomalies in four quarters of Europe and for the whole continent (the quarters are divided by the 45°N parallel and the 20°E meridian). In orange, the observations; in black the reconstruction with its shaded 95%-confidence interval; in blue the trend of the reconstruction and in red the trend of the observations.

Figure S11. Distribution of the correlations between each proxy and the reconstructed

temperature series. The proxies were sorted by decreasing order of maximum correlation (in absolute value) and only the proxies with significant correlations are presented. Two scales of correlations are used: for the first seven maps scale from -0.5 to 0.5 and for the others series, scale from -0.3 to 0.3 applies.

Table Captions

Table S1. Details and references of the proxies used

Table S2. Verification statistics for the estimation of 101 proxy data using the best analogues taken from the 1575-1744 reference period (the initial number of tree-ring series was 95, that of the historical series was 11, and that of the ice series was 8; 13 series were removed because insufficient number of data within the reference period) R is the correlation between estimates and observations on the reference period, Rp is the verification correlation on the data outside the reference period, RE is the reduction of error and CE the coefficient of efficiency (see text). A cross (+) indicates a confidence level of 75%, a star (*) 90%, two stars (**) 95% and three stars (***) 99%. These confidence levels are determined by Monte-Carlo simulation based 100 red noise series generated for each proxy series. Each random series follows an autoregressive order 1 process where the error term follows a Gaussian law and the order 1 autoregression term is that of the corresponding proxy series.

Table S3 - Variance of the 10 temperature principal components : "Total" line concerns the complete spectra and the three other ones the low-pass, middle-pass and high-pass bands. The three bands are delimited by the cut-off frequencies of 0.05 and 0.3 cycles/year.

Table S4 - Calibration and verification statistics for each frequency band of the 10 temperature PCs. R is the correlation between observations and estimates on reference data, Rp the corresponding correlation on independent data. R2 is the squared R. RMSEP is the root square of the mean error calculated on the independent data (h-block Jack-knife method, see text). RE is the reduction of error and CE the coefficient of efficiency, both calculated on the independent data.

Table S5 - Calibration and verification statistics for temperature series in each grid-point. R is the correlation between observations and estimates on reference data, R2 is the squared R. RE is the reduction of error calculated on the independent data. MPI95 is the 95% mean prediction interval, representing the one-side error bar for the reconstructions at the 95% level.

Table S6 - Correlations between each proxy and the 125 reconstructed temperature series (one for each grid-point): we have represented the minimum, mean and maximum of the 125 correlations and sorted them according to the maximum value.
