# Supplementary figures and images for: Growing Season Temperatures in Europe and Climate Forcings Over the Past 1400 Years
Source: PLoS One. 2010 Apr 1;5(4):e9972. doi: 10.1371/journal.pone.0009972 (PMC2848609; doi:10.1371/journal.pone.0009972)

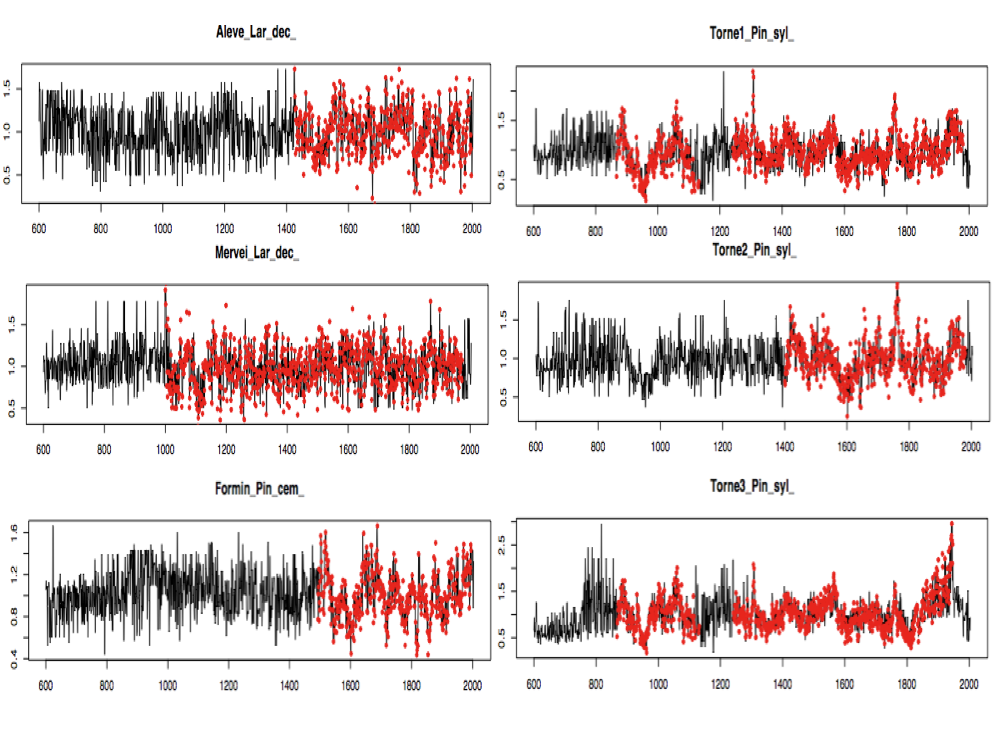

Supplement: Figure S1 — A few tree-ring series. Observations are displayed by red dots and estimates by black lines (estimates done by using the best analog method applied exclusively on the tree-ring series). (3.00 MB TIF) [file pone.0009972.s008.tif]

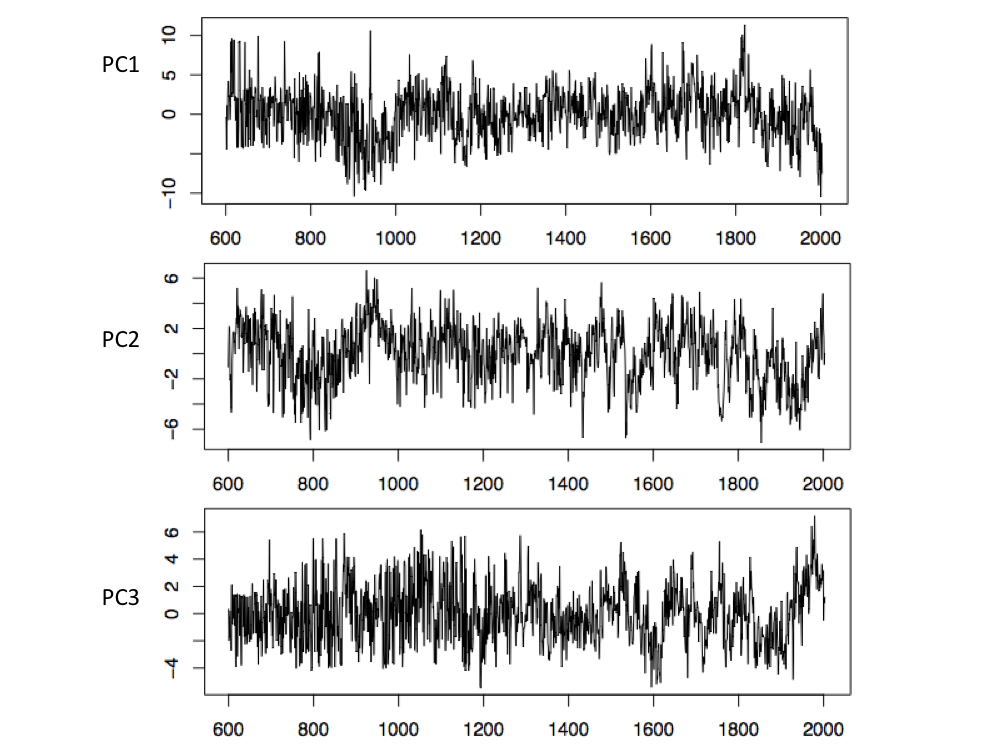

Supplement: Figure S2 — The first three principal components of the tree-ring series. (3.00 MB TIF) [file pone.0009972.s009.tif]

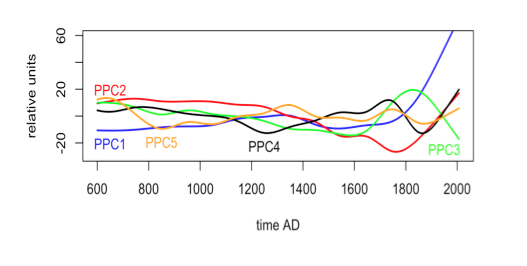

Supplement: Figure S3 — The first five principal components of the gridded annual temperature reconstructed from pollen data by [15]. (0.56 MB TIF) [file pone.0009972.s010.tif]

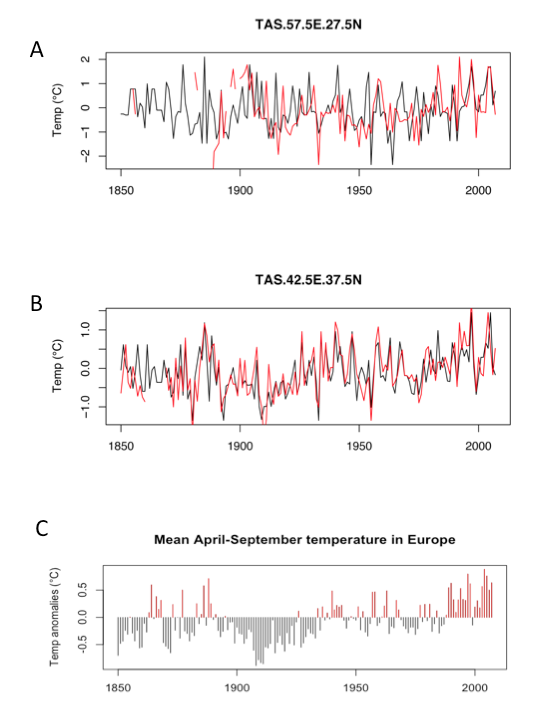

Supplement: Figure S4 — Mean April-September temperature in grid-points (27.5°N, 57.5°E) (A) (37.5°N, 42.5°E) (B) and averaged on the whole continent (125 series on 10°E to 60°W and 25°N to 75°N) (C). (1.58 MB TIF) [file pone.0009972.s011.tif]

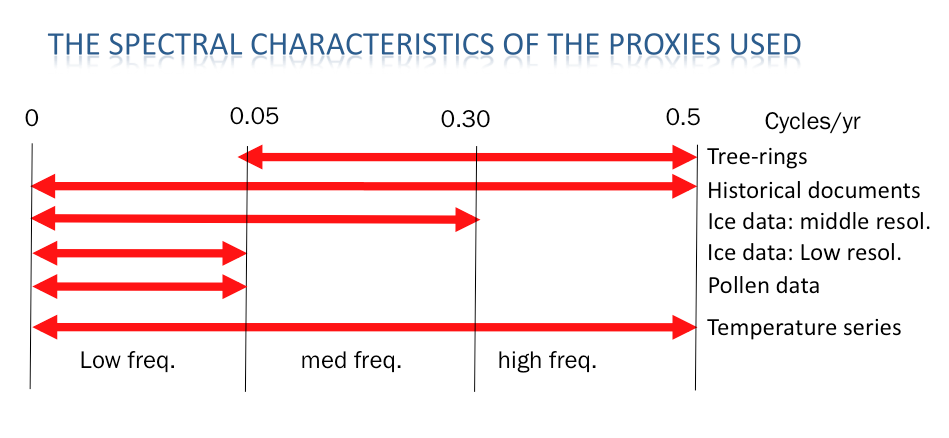

Supplement: Figure S5 — Scheme of the spectral characteristics of the proxies and temperature series: double red arrows indicate the frequency range of each proxy type in number of cycles per year. (1.66 MB TIF) [file pone.0009972.s012.tif]

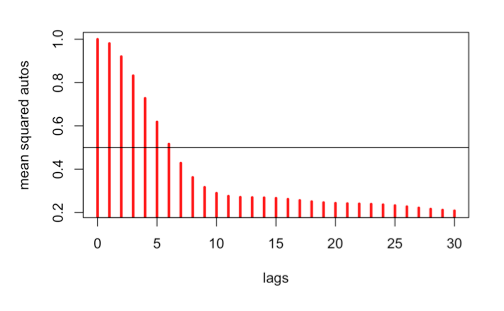

Supplement: Figure S6 — Proportion of variance in common between years separated by various lags from 0 to 30 (squared autocorrelation function). 0-lag represents the standardised variance, i.e. 1. The horizontal line represents the 50% variance. (0.63 MB TIF) [file pone.0009972.s013.tif]

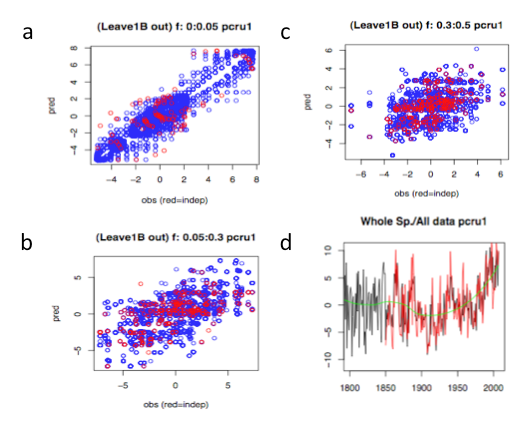

Supplement: Figure S7 — Estimates versus observations for the first PC of the HADCRUT3 April to September temperature series in the three frequency bands. Blue dots are data used for calibration and red dots data used for the h-block Jack-knife (leave 1-block out) verification. (a) in the low frequency domain, (b) in the middle frequency domain, (c) in the high frequency domain, (d) recombined estimated series (black) and observations (red) in function of time; the green line is the tendency. (0.92 MB TIF) [file pone.0009972.s014.tif]

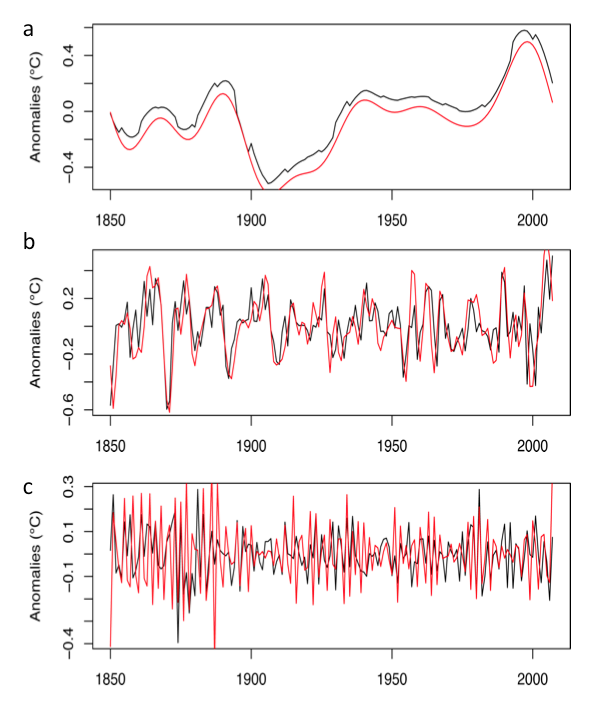

Supplement: Figure S8 — April to September temperature anomalies averaged on Europe: observation (red) versus estimates (black); (a) in the low frequency domain, (b) in the middle frequency domain, (c) in the high frequency domain. (1.73 MB TIF) [file pone.0009972.s015.tif]

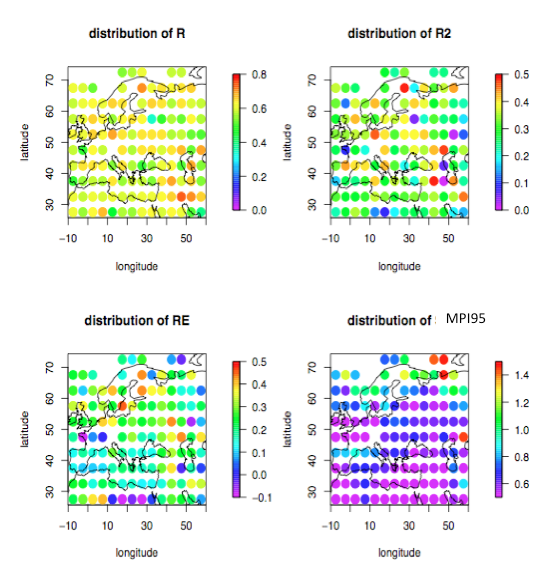

Supplement: Figure S9 — Spatial distribution of R, R2, RE and MPI95 (see text). (1.28 MB TIF) [file pone.0009972.s016.tif]

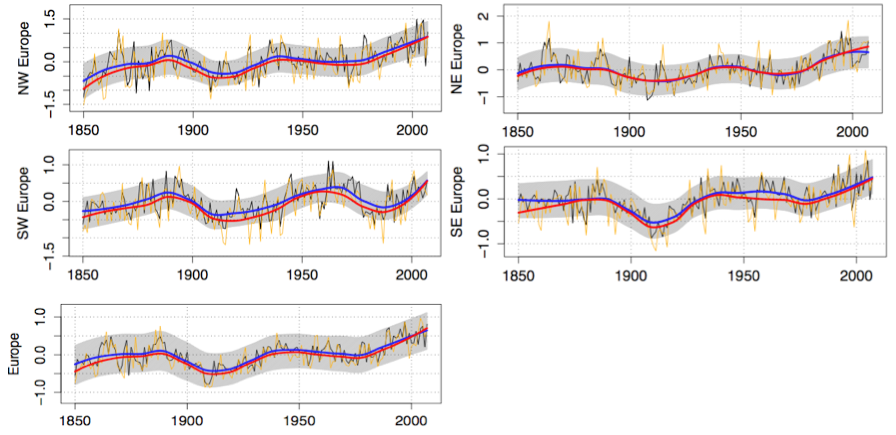

Supplement: Figure S10 — Comparison, on the reference period 1850–2007, of the reconstructed and observed April to September temperature anomalies in four quarters of Europe and for the whole continent (the quarters are divided by the 45°N parallel and the 20°E meridian). In orange, the observations; in black the reconstruction with its shaded 95%-confidence interval; in blue the trend of the reconstruction and in red the trend of the observations. (1.56 MB TIF) [file pone.0009972.s017.tif]

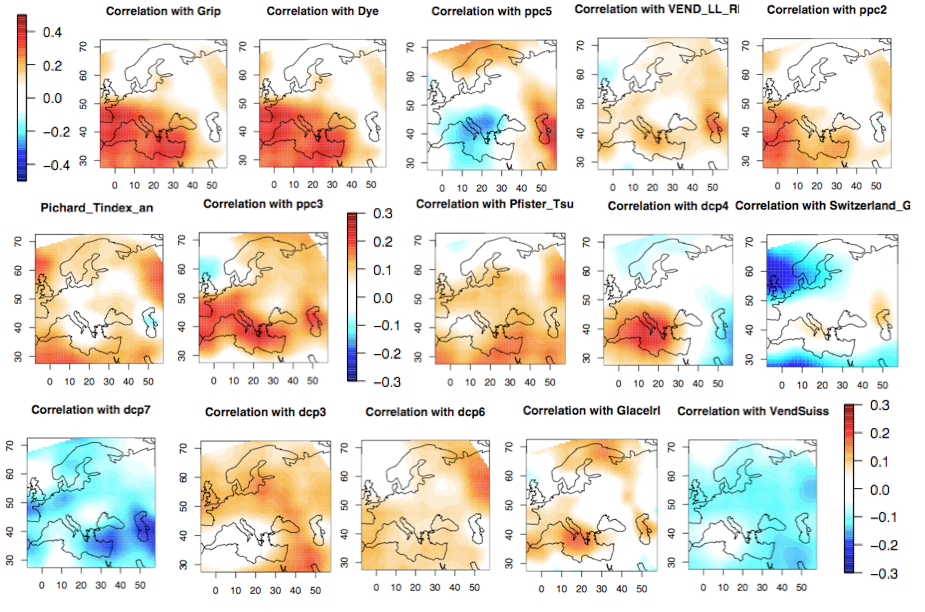

Supplement: Figure S11 — Distribution of the correlations between each proxy and the reconstructed temperature series. The proxies were sorted by decreasing order of maximum correlation (in absolute value) and only the proxies with significant correlations are presented. Two scales of correlations are used: for the first seven maps scale from −0.5 to 0.5 and for the others series, scale from −0.3 to 0.3 applies. (2.26 MB TIF) [file pone.0009972.s018.tif]
